# It's hard to keep all things angiogenic in one JAR!

**DOI:** 10.1186/2045-824X-3-1

**Published:** 2011-01-18

**Authors:** Jan Kitajewski, Yihai Cao, Mark Slevin

**Affiliations:** 1 Columbia University Medical Centre, Columbia New York, USA; 2 Karolinska Institute, Stockholm, Sweden; 3 ICCC, St Pau Hospital, Barcelona, Spain; 4 Manchester Metropolitan University, Manchester, UK

## It's hard to keep all things angiogenic in one JAR!

*Journal of Angiogenesis Research *has become "*Vascular Cell*".

"All things angiogenic" could mean many things to the many different types of researchers in the field. This diversity of thought is clearly reflected by the articles presented since the launch of the *Journal of Angiogenesis Research (JAR) *just over a year ago. Clinicians, scientists and educators have used the open access journal *JAR*, to present a variety of studies and concepts related to angiogenesis. Over the last year, *JAR *has presented reviews on Notch ligands found in vessels [1], Wnts in the brain vasculature [2], endothelial cell function of relevance to the cardiologist [3], and imaging paradigms to study and diagnose diseases of the vasculature [4]. Research reports on contraceptive-induced angiogenesis [5], on novel angiogenic regulators [6], and on capillary size and vascular permeability [7] represented some of the most highly accessed articles published in *JAR*. The field is large, diverse, exciting, and fast-paced. With notable ease, the field bridges the disciplines of medical diagnosis and treatment with that of fundamental scientific discovery. The field is relevant; serving as the basis of development of new drugs that are being used to treat cancer patients; those suffering from vision loss, diabetics, and those with other illnesses.

As a result of this diversity of thoughts, of research topics, and of researchers, we came to realize that all things angiogenic/vascular could not be kept in one single JAR! The publication platform, developed as a venue for angiogenic researchers, was found to be relevant and of interest to researchers studying the many facets of human vasculature, not limited to those who study the construction of new vessels. *Journal of Angiogenesis Research *has grown into new areas and new frontiers of research. Our stated aim was "to publish articles from all areas of the 'broad spectrum' of vascular research and from the bench to the bed-side" (September 2009). To best achieve this aim, we here present a new name for the journal, *Vascular Cell*.

*Vascular Cell *will focus on the vasculature in health and disease. Appreciated by the earliest anatomists and physicians, the vasculature is now being redefined. The vasculature is a dynamic structure that is built and rebuilt. Although early researchers focused on endothelial cells and started with a limited knowledge of "perivascular" cells, the vasculature is built from a wide variety of cell types and influenced by an even wider array of cells. The last decade of vascular research has been exemplified by a wider appreciation of what the vasculature is and of what it is made up of. We learned that endothelial cells come from several sources, even pathologically mutated cells! We learned that the human vasculature can be influenced by immune cells, nervous system cells, endocrine cells; in short, cells from all types of tissues. Contemporary research seeks to understand how these diverse cell types integrate signals to generate a functional vasculature. Diseases of the vasculature will be better understood, and hopefully better treated, by studying all cells of the vasculature. In the spirit of this broader definition of the vasculature, the new name represents an essentially broader focus for the journal. We hope that *Vascular Cell *serves as the venue to present timely research and thought on the multi-faceted vasculature in the 21^st ^century.

Vascular biology including angiogenesis research is one of the fastest expanding fields in biomedical research. Almost all developmental processes are coupled to vascular growth, remodeling and functions. Structural or functional defects of the vascular network are tightly linked to the onset, development and progression of various human diseases including those most common and lethal disorders such as cancer, cardiovascular disease, obesity, metabolic disorders and chronic inflammation. While major scientific journals such as *Cell *and *Nature *series cover other rapidly developing fields, including immunology, neuroscience, stem cell, cancer and infection, vascular biology has not been highlighted as a major field standing on its own. Our goal is to establish *Vascular Cell *as one of the top journals to publish the best quality research and review articles in this field. Importantly, we hope that this journal will further boost research interests from other fields in connection to vascular biology. We also emphasize the translational research bridging basic research to clinical implications and vice versa. As several FDA-approved anti-angiogenic drugs are in clinical use for the treatment of malignant and non-malignant diseases, vascular translational research will add unprecedented values to clinical practice of these targeted drugs. We are aiming to set up the highest standard for publishing primary research, translational research, clinical, methodological, case reports and review articles.

Specific examples of this overlapping/novel technology include recent developments in tissue engineering, where human fibroblasts extracted from skin biopsies were formed into a cohesive sheet and then used as a matrix to engineer new blood vessels using Teflon-coated stainless steel support tubes and attachment of an internal membrane and peripheral adventitial coating using patient-only cells. These have been shown to be antithrombogenic and stable for extended periods of time and could provide a valuable alternative to the use of synthetic or xenogeneic vessels for transplant in patients with advanced cardiovascular disease [8]. Nanotechnology will have an increasing role in development of scaffolding, targeting of molecules and imaging platforms over the next few years. Optimizing the process of vascularization/angiogenesis is important in the treatment of ischaemic disease, and Sinha Roy et al [9] demonstrated this recently by encapsulating NK1 (a splice variant of hepatocyte growth factor) in biodegradable nanoparticles composed of D, L-lactic acid-co-glycolic acid copolymer, and demonstrated both temporal release and enhanced vascularization in a matrigel-implanted zebrafish model of vasculogenesis.

In other vascular diseases, including neurodegenerative diseases such as Alzheimer's, understanding the mechanisms that cause vascular abnormalities and impair cerebral blood flow could provide some of the answers to the importance of ischaemia (possibly initiated following ischaemic or lacunar stroke) in formation of vascular amyloidosis and dense-core plaques [10]. Newly developed and currently developing vascular imaging techniques may help us to identify earlier, patients at risk of progressing to pre-senile dementia, and also could be useful in detecting vascular-associated lesional changes after stroke, and in primary (e.g. glioblastoma) or metastasized brain tumours. In this respect, Kienast et al [11] used multiphoton laser scanning microscopy to image single steps of metastasis formation in a mouse brain in real-time. Translated to humans, understanding the individual fate of tumour cells, their movement through microvessels and perivascular growth could help us modify existing therapeutic strategies. These are only a few examples of the range of topics appropriate to be included in *Vascular Cell*. A review of the processes of angiogenesis and vascularization in all the major diseases is presented in the recent book published recently by Springer entitled ("Therapeutic angiogenesis", Molecular Mechanisms and Targeted Clinical Approaches for the Treatment of Angiogenic Disease [12].
